# Radial Peripapillary Capillary Vessel Density as a New Biomarker in Pseudophakic Cystoid Macular Edema

**DOI:** 10.3390/jcm14051454

**Published:** 2025-02-21

**Authors:** Michele Rinaldi, Gilda Cennamo, Marina Concilio, Alessandro Aurilia, Antonio Alfano, Emilia Chiara Russo, Ciro Costagliola

**Affiliations:** 1 Department of Neurosciences, Reproductive Sciences and Dentistry, University of Naples “Federico II”, Via Sergio Pansini 5, 80131 Naples, Italy; michrinaldi@libero.it (M.R.); conciliomarina@gmail.com (M.C.); ale.aurilia3@gmail.com (A.A.); antonio.alfano@live.com (A.A.); emiliachiararusso@gmail.com (E.C.R.); ciro.costagliola@unina.it (C.C.); 2 Department of Medicine and Health Science V. Tiberio, University of Molise, 86100 Campobasso, Italy

**Keywords:** phacoemulsification, cystoid macular edema, optical coherence tomography, optical coherence tomography angiography, radial peripapillary capillary plexus

## Abstract

**Background/Objectives:** Our aim was to investigate the vessel density (VD) of the radial peripapillary capillary (RPC) plexus in eyes with early pseudophakic cystoid macular edema (PCME) and controls using OCT angiography (OCTA). **Methods**: Patients with PCME and controls underwent structural OCT to assess the retinal nerve fiber layer (RNFL) and central macular thickness (CMT) and 6 × 6 mm^2^ macular OCTA to record the superficial (SVP), deep (DVP) vascular plexus, and choriocapillaris. A scan area of 4.5 × 4.5 mm^2^ was centered on the optic disk to analyze the RPC plexus VD. Fluorescein angiography was performed in PCME patients. **Results**: Data from 30 PCME and 30 control eyes, matched for age, were analyzed. The mean CMT was larger in PCME eyes than in control eyes (450 ± 6.5; 243 ± 3.5 micron, *p* < 0.001). The mean RNFL thickness was significantly greater in terms of global thickness in the PCME group compared to the control group (103 ± 5.2; 91 ± 2.5 micron, *p* < 0.001). The PCME group had a statistically significant increase in the VD of the RPC in the whole image, peripapillary region, and inside disk compared to the control group (*p* < 0.001). There was no difference in SVP (*p* = 0.660) or DVP (*p* = 0.480) VD between the two groups. A significant correlation was found between the average RNFL thickness and the VD of the RPC (*p* < 0.05). **Conclusions**: Eyes with PCME showed increased VD of the RPC and a correlation between this parameter and RNFL thickness; the VD of the RPC shows potential as a reliable non-invasive biomarker for the early diagnosis of PCME.

## 1. Introduction

Pseudophakic cystoid macular edema (PCME), also known as Irvine–Gass syndrome (IGS), is an accumulation of fluid in the macula that occurs after cataract surgery, with an early or late presentation (cut-off 3 months) [1]. It is the most common cause of decreased vision after uneventful phacoemulsification, with a rare incidence of 0.1–2.35% for clinically significant PCME [2].

Pseudophakic cystoid macular edema (PCME) is the most common cause of the deterioration of visual acuity after uncomplicated cataract surgery. It is also known as Irvine–Gass syndrome (IGS) and is characterized by fluid accumulation, both intra- and subretinal. The onset is variable and is defined as early or late, with a cut-off of 3 months [1,2].

To date, its pathophysiology has not been clearly elucidated, although several factors such as vascular retinal pathologies (diabetes, retinal vein occlusion, uveitis) and intraoperative complications (posterior capsule rupture) have been identified as possible risk factors for PCME [3]. The main trigger is thought to be surgical trauma to the intraocular tissue of the anterior segment of the eye, in which the blood–aqueous barrier is broken. 

As a result, an inflammatory cascade is triggered: the presence of pro-inflammatory molecules increases in the vitreous cavity, the blood–retinal barrier breaks down, and there is progressive leakage from the retinal capillaries and the accumulation of extracellular fluid in the retina. The outer plexiform layer (OPL) and the inner nuclear layer (INL) are the retinal layers most involved in the process [4,5].

These progressive architectural changes have been thoroughly described by Sigler EJ and colleagues using optical coherence tomography (OCT), an in vivo non-invasive technique. They demonstrated, by longitudinal analysis, that PCME starts with cystic changes in the INL, progresses to OPL involvement, and may continue to subretinal fluid [6]. OCT is a fundamental tool for the diagnosis, follow-up, and morphological assessment of patient response to treatments.

Nevertheless, fluorescein angiography (FA) still represents the gold standard with which to perform differential diagnosis for macular edema because it is sensitive to leakage from vessels. Indeed, FA identifies early perifoveal capillary dilatation and leakage with the pooling of fluorescein into macular cystoid spaces in a stellate or rosette perifoveal pattern and highlights the hyperfluorescence of the optic disk in late phases, which is a hallmark of IGS diagnosis [7].

With the increasing implementation of the technology, OCT angiography (OCTA), a non-invasive modality of vascular examination, has proven its role in the investigation of retinal vascular diseases from both clinical and research perspectives [8]. OCTA allows the direct visualization of retinal and choroidal microvasculature and has increasingly improved the understanding of retinal involvement in ocular and systemic diseases [9,10].

Moreover, FA is dominated by the superficial vascular plexus, whereas OCTA can visualize and analyze the radial peripapillary (RPC) and deep capillary networks [11]. Several research groups have investigated vascular retinal changes in the macula that are associated with PCME [1,12].

To our knowledge, this is the first study to investigate possible abnormalities in the vascular network of the optic nerve head in patients with IGS compared to healthy eyes, using OCTA, in order to better understand the vascular pathophysiological mechanisms involved in this disease.

## 2. Materials and Methods

In this observational prospective study, 60 eyes from 60 patients were studied from July 2023 to December 2023 at the Eye Clinic of the University of Naples “Federico II”. The study was registered in Clinicaltrial.gov (NCT06436170), conducted in compliance with the tenets of the Declaration of Helsinki, and approved by the Ethics Committee of the University of Naples Federico II (No. EC-22/2024) on July 2024. Written informed consent was obtained from all participants before enrollment, with strict adherence to confidentiality and privacy standards.

### 2.1. Patients

Overall, 30 eyes of 30 patients included in the study were affected by IGS and 30 healthy subjects represented the control group. All patients and controls underwent a complete ophthalmological examination, including the best-corrected visual acuity (BCVA) evaluation, intraocular pressure (IOP) assessment with Goldmann applanation tonometry, biomicroscopy, fundus examination, spectral domain–OCT (SD-OCT), OCTA (RTVue XR Avanti, Optovue, Fremont, CA, USA), and fluorescein angiography (Heidelberg Engineering, Heidelberg, Germany). All patients with a diagnosis of PCME following phacoemulsification surgery, addressed to our clinic, were enrolled in the study.

**Figure 1:**
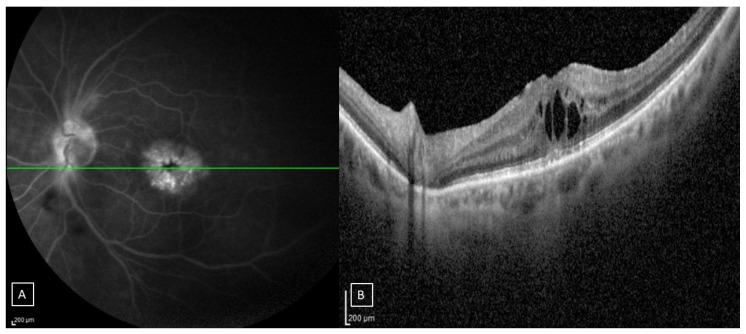
(**A**): Fluorescein angiography results of a patient’s left eye showing perifoveal petaloid pattern with late leakage of the optic disk; (**B**): horizontal SD-OCT B-scan passing to the fovea area (green line) of the same eye showing macular thickening due to intraretinal cystoid spaces.

The diagnostic criteria for PCME were as follows: (1) vision loss in the operated eye within 3 months after surgery in patients with acute PCME; (2) changes in B-scan structural OCT, with elevated foveal contour, intraretinal cystoid spaces, and a central macular thickness (CMT) of at least 300 micron; (3) the leakage and pooling of dye in macular cystoid spaces and late hyperfluorescence of the optic nerve head on FA. Clinical suspicion of PCME was confirmed using FA and structural OCT (Figure 1).

Inclusion criteria were as follows: age > 18 years; non-complicated cataract surgery; a diagnosis of PCME; and confirmation by FA, OCT, and macular and papillary OCTA. Exclusion criteria were as follows: known previous retinal diseases (diabetes, vein occlusion, uveitis, vasculitis, age-related macular degeneration, hereditary macular dystrophy, and glaucoma) and any other maculopathy; a history of intraocular surgery, vitreoretinal and retinal vascular diseases, uveitis, and congenital eye disorders; myopia > 6 diopters; and low-quality images obtained with SD-OCT and OCTA.

### 2.2. Spectral Domain Optical Coherence Tomography

Mean circumpapillary RNFL and central macular thickness (CMT) were measured (software RTVue XR version 2017.1.0.151, Optovue Inc., Fremont, CA, USA). The optic nerve head map protocol was used to evaluate the circumpapillary RNFL on measurements obtained around a circle, 3.45 mm in diameter, centered on the optic disk [13]. Only high-quality images, as defined by a signal strength index above 40, were accepted. All images were analyzed by two trained examiners, who rejected scans that had motion artefacts, poor centration, incorrect segmentation, or poor focus.

### 2.3. Optical Coherence Tomography Angiography

OCTA images were obtained using the RTVue XR Avanti, Optovue, Inc. (software RTVue XR version 2017.1.0.151, Freemont, CA, USA) following a standardized protocol based on the split-spectrum amplitude de-correlation algorithm, as previously described [13]. The macular capillary plexus was visualized performing a 6 × 6 mm^2^ scan over the macular region, centered on the fovea, and the percentage area occupied by the large vessels and microvasculature in the analyzed region defined the vessel density (VD) [14]. The software identified the VD in whole area of the macular scan, considering the two retinal vascular networks, namely, the superficial and deep capillary plexuses (SCP, DCP) and the choriocapillaris. The Angio–Vue disk mode automatically segmented the radial peripapillary capillary (RPC) plexus VD, analyzing the whole papillary region with a scanning area of 4.5 × 4.5 mm^2^ centered on the optic disk. VD was automatically calculated for the radial peripapillary capillary plexus in the superficial retinal layers. This extended from the inner layer membrane to the retinal nerve fiber layer posterior boundary [15]. When segmentation errors were recorded in Angio–Vue automated mode acquisition, image segmentation was manually adjusted by one of senior authors (G.C.). Working according to the recent literature, the SCP included a slab extending from the internal limiting membrane to the inner plexiform layer (IPL) and the DCP included a slab extending from the IPL to the outer plexiform layer (OPL) [16]. All images were analyzed by two trained examiners.

In case of a low-quality image due to eye movements, an image was discarded and the acquisition was repeated. The images with a final signal strength index < 70 were excluded from the analysis [17].

### 2.4. Statistical Analysis

Statistical analysis was performed with the Statistical Package for Social Sciences (Version 20.0 for Windows; SPSS Inc., Chicago, IL, USA). The chi-squared test was used to determine differences in terms of sex among groups. One-way analysis of variance (ANOVA) followed by Bonferroni post hoc analysis was used to separately evaluate differences for the OCT and OCTA parameters among the study groups.

## 3. Results

A total of 60 eyes from 60 subjects, of whom 30 were IGS patients (14 females, 16 males, mean age 72.29 ± 7.05 years), and 30 were healthy subjects (13 females, 17 males, mean age 72.66 ± 7.05 years), were included in this observational study. There were no statistically significant differences in age (*p* = 0.859) and sex (*p* = 0.380) among the groups (Table 1).

At OCT scans, IGS patients showed an increase of CMT (mean ± SD: 450 ± 6.5 micron) with respect to the control group (mean ± SD: 243 ± 3.5 micron, *p* < 0.001). In terms of RNFL measurement, the IGS group had greater global thickness (mean 103 ± 5.2 micron) with respect to the control group (mean ± SD: 91 ± 2.5 micron, *p* < 0.001 (Table 2).

**Figure 2:**
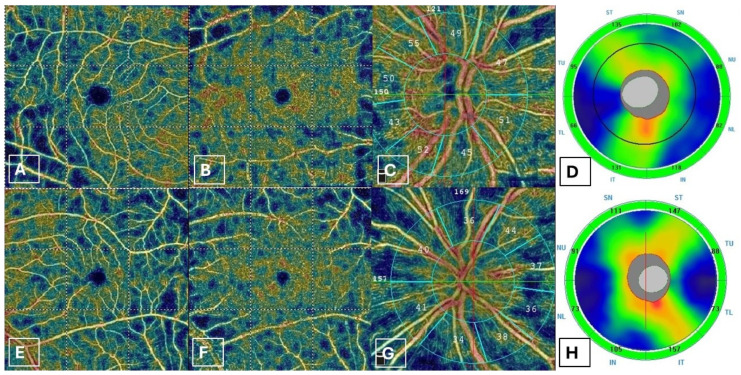
The right eye (**A**–**D**) of a patient with PCME diagnosis from the study group shows normal vessel density in superficial and deep capillary plexuses at OCTA (**A**,**B**), and increased vessel density in the radial peripapillary capillary plexus at OCTA (**C**). SD-OCT shows increased global RNFL thickness (101 micron) (**D**). The left eye (**E**–**H**) of a healthy patient of the control group shows normal vessel density/VD in superficial, deep retinal capillary plexuses, and normal vessel density in radial peripapillary capillary plexus at OCTA (**E**–**G**). SD-OCT shows normal global RNFL thickness (96 micron) (**H**).

Upon OCTA examination, IGS patients exhibited a statistically significant increase in the VD of RPC with respect to controls in the whole image (*p* = 0.001). There were no statistically significant differences in SCP (*p* = 0.660) and DCP (*p* = 0.480) among the groups. A *p* value (*p* < 0.001) was considerably statistically significant (Figure 2).

Finally, we evaluated the relationships between the RNFL and VD of RPC parameters in IGS patients. We found a significant correlation between the RNFL average and the VD of RPC (*p* < 0.05) (Table 3). Interestingly, both the RNFL and VD of the RCP are increased in patients with early PCME, suggesting a potential link between vascular and structural features in this pathology.

## 4. Discussion

This study is the first to investigate the structural and microvascular changes in the papillary region in IGS patients to better understand the pathophysiological mechanisms of this disease. Our results demonstrated a significant increase in RNFL and RCP in the study group with respect to controls, while no significant difference was found in the comparison between two groups in either SCP or DCP.

Pseudophakic cystoid macular edema (PCME) is a benign condition characterized by the presence of fluid in the layers of the macula, specifically in the inner nuclear layer and in the Henle’s fiber layer, and usually presents within a few weeks after uneventful cataract surgery [18]. It is the most common cause of reduced visual acuity after this type of surgery.

Regarding pathophysiology, some authors have hypothesized an iatrogenic inflammatory trigger, starting in the anterior segment of the eye, leading to a breakdown of the blood–retinal barrier and the upregulation of pro-inflammatory proteins in the retina [19,20]. Recently, Predovic J. et al. suggested a possible correlation between the detection of hyperreflective spots in the vitreous and the increase in central macular thickness, as this finding would explain the remnants of lens fragments formed during the phacoemulsification phase [21]. Spaide and colleagues have suggested that cystoid macular edema may be associated with two phenomena: ischemia and inflammation. In both cases, the most affected plexus is the DCP, at the level of which vascular flow is reduced, so it is not just a matter of vascular displacement. In diabetic CME, ischemic phenomena predominate, unlike in IGS, where changes in the VD of the DCP have been shown to improve when the edema itself disappears [22].

There are no specific guidelines for treatment in the literature; therefore, the theoretical background, clinical experience, and safety of the procedure are described as the elements to be considered in order to choose the most appropriate treatment in relation to the clinical entity [23].

Moreover, PCME resolves spontaneously or with pharmacologic treatments such as non-steroidal anti-inflammatory drugs NSAIDs and/or carbonic anhydrase inhibitors [24]. However, it is known that some cases do not heal with these medications and need to be supported by periocular or intravitreal glucocorticoids, which occurs with success [25,26]. Finally, Verdina et al. investigated the potential use of a subthreshold micropulse yellow laser in refractory PCME and described an excellent anatomical outcome with the complete resolution of retinal edema [27]. Despite various topical treatments and anatomical restorations, up to 27% of eyes do not achieve visual *restitutio ad integrum* due to subtle changes in the morphology of the outer photoreceptors [28].

In addition to clinical assessment, the diagnostic evaluation of PCME patients requires retinal imaging. Nowadays, fluorescein angiography (FA) is still the gold standard with which to detect the typical petaloid perifoveal pattern and the hyperfluorescence of the optic disk in the late phase. Although FA identifies leakage flowing into the cystoid retinal spaces, structural OCT is necessary to accurately delineate the anatomic location of edema in the retinal layers. Recently, Rabina et colleagues suggested RNFL thickness as a possible additional parameter for the diagnosis and follow-up of PCME, as they showed that patients with PCME have an increase in RNFL thickness compared to healthy eyes and that this parameter becomes thinner after PCME subsides [29]. Rabina, in agreement with Hwang’s published data, argued that the increase in RNFL thickness in a sectoral and then diffuse manner is linked to the presence of macular edema and its rapid development [30].

To date, several research groups have published data on macular vascular changes in patients with IGS. Chetrit et al. described a significant decrease in vessel density (VD) in both vascular plexuses, which was greater in the DCP group than in the SCP group compared to healthy control eyes; the morphological pattern remained almost normal in the SCP and was disorganised in the DCP [1]. At the same time, Sacconi and colleagues also demonstrated both functional and anatomical changes in the DCP [12].

OCTA investigation can be related to several elements of limitation, such as low signal strength, false signals generated by eye movements, segmentation errors, projection, and shadowing artifacts [31].

Of course, these limitations must be considered when performing OCTA, and a critical approach is required when analyzing the images. To overcome these limitations, we excluded all low-power images in our study, we used automatic segmentation mode, with manual segmentation if necessary, and all images were analyzed by two trained examiners.

Although vascular the abnormalities of the macula were thoroughly studied, to the best of our knowledge, this is the first study that evaluates, using OCTA, the characteristics of retinal vascular plexuses of the optic disk head in IGS patients in comparison to healthy eyes.

Our results highlighted, for the first time, the increase in the VD of RPC in the whole image, specifically affecting the peripapillary region in PCME patients. The increase in vessel density of the RPC in PCME patients could be related to an inflammatory trigger that starts in the anterior segment of the eye, leading to a breakdown of the blood–retinal barrier and the upregulation of pro-inflammatory proteins in the retina. Indeed, according to our results, both RNFL and VD of the RCP are increased in patients with early PCME. These data are another building block for the inflammatory hypothesis about the nature of IGS. If, as Rabina hypothesises, the increase in RNFL thickness is related to the presence of macular edema, the increase in VD cannot be explained by a simple mechanical effect. Moreover, the increase in VD of the RCP could be caused by the angiographic appearance in OCTA of late hyperfluorescence of the optic disk, a phenomenon associated with a vascular inflammatory component. Furthermore, as previously shown in the literature, the chronic thickening of the RNFL plays a role in visual acuity deterioration, and so the restoration of RNFL thickening and the RNFL structure is valuable for ensuring visual acuity after therapy [30].

The role of RCP in clinical practice was not the topic of this study. However, the results suggest that RCP could be an interesting non-invasive parameter to consider in the diagnosis and follow-up of IGS. Although not all patients diagnosed with IGS have visual symptoms, Dabas and colleagues showed that postoperative OCT examination reveals some degree of macular edema [32]. Postoperative OCTA and OCT might reveal a low level of inflammation and be useful in assessing potential patients who might benefit from prolonged postoperative treatment in order to reduce the risk of subsequent IGS.

This study has several limitations. First, the sample size is small in relation to the low incidence rate of PCME after mini-invasive phacoemulsification surgery and the inclusion criteria we defined. Indeed, all the included patients underwent uneventful cataract surgery and were sent to our Ophthalmology Unit to undergo fluorescein angiography, in relation to decreased postoperative visual acuity [33]. Secondly, the absence of a longitudinal follow-up is a limitation.

Longitudinal studies with larger sample sizes should be planned, focusing on patients’ baseline in the preoperative period, defining a standard of care after surgery, and evaluating follow-up of at least 6 months for those who develop IGS. These studies could help us to determine the potential role of the preoperative VD of RCP as a predictive biomarker for the development of IGS, its variation during follow-up, and its possible correlation with visual recovery. The longitudinal assessment of VD RCP in the follow-up of patients with IGS could be interesting to evaluate the retinal anatomical vascular response to topical treatment.

## 5. Conclusions

In conclusion, the use of OCTA as a diagnostic tool for IGS may be useful to change the management of patients with IGS, as it may allow clinicians to switch from an invasive diagnostic exam such as FA to a non-invasive one, which has a lower risk of adverse effects and better availability. RPC vessel density could play a role from both a scientific and clinical perspective. Indeed, it could be a helpful and meaningful biomarker for identifying early microvascular changes in the retina to better understand the pathophysiologic mechanisms of this disease and the change in vascular abnormalities during treatment. RCP vascular density could play a potential role as a biomarker at different stages of the management of patients with IGS, from diagnosis to treatment. Future longitudinal studies will help us to better define its role.

## Figures and Tables

**Table 1:** Demographic characteristics of PCME and control groups.

	Study Group	Control Group	*p*-Value
**Eyes**	30	30	-
**Female/Male**	14/16	13/17	0.859
**Age (mean age ± SD, years)**	72.29 ± 7.05	72.66 ± 7.05	0.380
**BCVA (logMAR)**	0.09 ± 0.09	11 ± 0.09	0.001
**IOP**	10 ± 5.2	12 ± 2.5	0.901

Data are expressed as mean ± SD. PCME: pseudophakic cystoid macular edema; BCVA: best-corrected visual acuity; logMAR: logarithm of the minimum angle of resolution; IOP: intraocular pressure.

**Table 2:** Differences in OCT angiography and SD-OCT parameters between PCME (pseudophakic cystoid macular edema) group and healthy subjects.

			Study Group	Control Group	ANOVA *p*-Value
**OCTA**	**SCP (%)**	Whole image	49.95 ± 5.17	51.99 ± 2.52	<0.660
**parameters**	**DCP (%)**	Whole image	55.82 ± 8.07	54.83 ± 5.46	<0.480
	**RCP (%)**	Whole image	56.22 ± 3.07	53.13 ± 5.46	<0.001 *
	**CMT average (µm)**		450 ± 6.5	243 ± 3.5	0.001 *
	**RNFL average (µm)**		103 ± 5.2	91 ± 2.5	0.001 *

Data are expressed as mean ± SD. SCP: superficial capillary plexus: DCP: deep capillary plexus; RCP: radial peripapillary capillary plexus; CMT: central macular thickness; RNFL: retinal nerve fiber layer. Student’s *t*-test for independent samples and analysis of variance (ANOVA) followed by Bonferroni post hoc analysis. * Statistical significance *p*-value < 0.001.

**Table 3:** Demographic characteristics of PCME and control groups.

			Study Group	Control Group	ANOVA *p*-Value
**OCTA**	**SCP (%)**	Whole image	49.95 ± 5.17	51.99 ± 2.52	<0.660
**parameters**	**DCP (%)**	Whole image	55.82 ± 8.07	54.83 ± 5.46	<0.480
	**RCP (%)**	Whole image	56.22 ± 3.07	53.13 ± 5.46	<0.001
	**CMT average (µm)**		450 ± 6.5	243 ± 3.5	0.001 *
	**RNFL average (µm)**		103 ± 5.2	91 ± 2.5	0.001 *

Data are expressed as mean ± SD. SCP: superficial capillary plexus; DCP: deep capillary plexus; RPC: radial peripapillary capillary plexus; CMT: central macular thickness; RNFL: retinal nerve fiber layer. Student’s *t*-test for independent samples and analysis of variance (ANOVA) followed by Bonferroni post hoc analysis. * statistical significance *p*-value < 0.001.

## Data Availability

Data are available on reasonable request.
